# Metastatic gallbladder adenocarcinoma with signet-ring cells: A case report

**DOI:** 10.1186/1752-1947-5-458

**Published:** 2011-09-14

**Authors:** Fernando Bazan, Juan Sanchez, Guadalupe Aguilar, Aleksandar Radosevic, Marcos Busto, Flavio Zuccarino, Lara Pijuan, Noelia Risueño

**Affiliations:** 1 Department of Radiology, Parc de Salut Mar Hospital, Barcelona, Cataluña, Spain; 2 Department of Pathology, Parc de Salut Mar Hospital, Barcelona, Cataluña, Spain

## Abstract

**Introduction:**

Signet-ring cell carcinoma is a rare and aggressive variant of mucinous adenocarcinoma. Only a few cases of gallbladder adenocarcinoma with signet-ring cells have been reported and because of this there is a lack of knowledge about the behavior and biology of this pathology.

**Case presentation:**

We present the case of a 63-year-old Arab man with gallbladder signet-ring cell adenocarcinoma. He had an elective cholecystectomy and refused chemotherapy. Two months later, a small hepatic metastatic nodule was found, and nine months later he presented with multiple metastases in the liver, lymphatic nodes, both pleuras, peritoneum and subcutaneous tissue.

**Conclusion:**

The proliferation of signet-ring cells in a gallbladder adenocarcinoma worsens the prognosis of an already adverse neoplasm. New lines of treatment in chemotherapy, such as cisplatin, or new biological therapy, such as monoclonal antibody c-myc oncogene, should be encouraged to improve the survival and life quality of these oncologic patients.

## Introduction

Gallbladder carcinoma (GC) is the fifth most common malignant tumor of the gastrointestinal tract and the most frequent malignant neoplasm of the biliary tract [1].

Approximately 99% of gallbladder cancers are carcinomas including 90% of adenocarcinomas, mostly well or moderately differentiated (74%). Five percent of gallbladder carcinomas comprise other subtypes such as papillary adenocarcinomas, squamous cell carcinomas and mucinous adenocarcinomas [1].

Signet-ring cell carcinoma (SRCC) is a rare and aggressive variant of mucinous adenocarcinoma. It is histologically characterized by the presence of rounded cells with a clear and mucinous cytoplasm and a peripheral nucleus. Its aggressive behavior is shown by the infiltration of the surrounding stroma, broad dissemination and a high tendency to produce peritoneal metastases in the gastrointestinal tract, as in our patient [2].

The presence of non-neoplastic signet-ring cells on normal tissues is a source of pitfalls in biopsy specimens that leads to over-diagnosis of SRCC. Although the meaning of this histological finding is still unclear [3], the features that define this entity are: the confinement of non-neoplastic cells to the mucosal surface, their lack of cellular atypia [4] and necrotic changes with surrounding inflammation [5].

GC-related symptoms are nonspecific. The risk factors have not been determined yet, although a close relationship with gallstones has been described [6]. As a result, almost one percent of all cholecystectomies have been reported to contain a malignant neoplasm focus [7].

## Case report

A 63-year-old Arab man with symptoms of three-month duration including a dull epigastric pain radiating to the right hypochondrium was transferred from a local hospital to our University Hospital. An upper endoscopy was performed and a mild gastritis (*Helicobacter pylori *negative) was diagnosed. The patient received proper treatment, but the pain persisted. Ultrasonography revealed many gallstones with thickened wall of the gall bladder. Images were not available to us because these examinations were performed before the referral of the patient.

He was scheduled to undergo an elective cholecystectomy in our center. According to his medical history he had frequent episodes of biliary colic and a cholangitis episode which resolved following endoscopic retrograde cholangiopancreatography papillotomy (ERCP). He had a normal cholangioresonance study.

Laparoscopic cholecystectomy revealed an empyema of the gallbladder with stones which made the dissection very difficult because of local inflammation. The gallbladder was finally removed inside a vinyl extraction bag.

The day after surgery, the patient complained of pain at the right hypochondrium and a 2 g hemoglobin decrease was detected. Computed tomography (CT) showed a perihepatic hematoma extending to the right paracolic gutter and no suspicious focus of other neoplasm was found.

**Figure 1:**
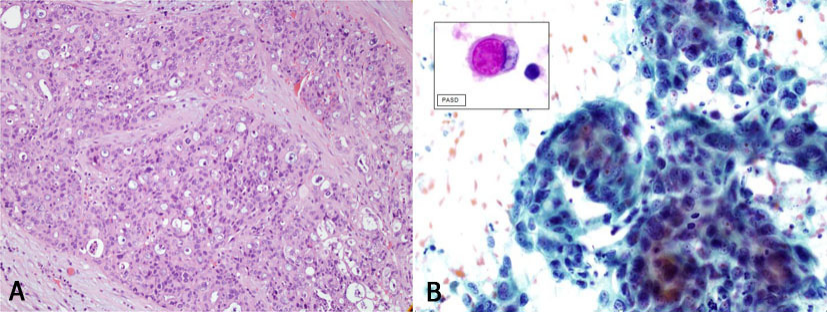
**A: Poorly differentiated gallbladder adenocarcinoma with signet-ring cells (Hematoxylin- eosin, original magnification ×20)**. **B**: High power magnification and inset: Signet-ring cell with cytoplasmatic vacuole of mucinous material. (Diastase-PAS stain).

Histology revealed a poorly differentiated adenocarcinoma with signet-ring cells (SRCC) extending to the surrounding connective tissue, as well as to the microvasculature and invasion of the cystic duct surgical margins (Figure 1). Immunohistochemical staining showed p53 mutation and CK7 were positive; results for CK20 and estrogen were negative. He was histologically classified as a grade 3 (poorly differentiated) with a T2NxMx stage. After his recovery, a radical resection and chemotherapy was proposed, but he rejected this treatment for personal reasons.

Several CTs and ultrasound scans were performed as follow up of the hematoma, and no images of widespread disease or other complications were found until seven weeks after surgery, when a CT revealed a 19 mm hypodense nodule on the VIII liver segment. We performed a fine needle aspiration (FNA) to evaluate this nodule. It was shown on cytology to be a metastatic SRCC. After a new evaluation, the patient rejected chemotherapy again.

**Figure 2:**
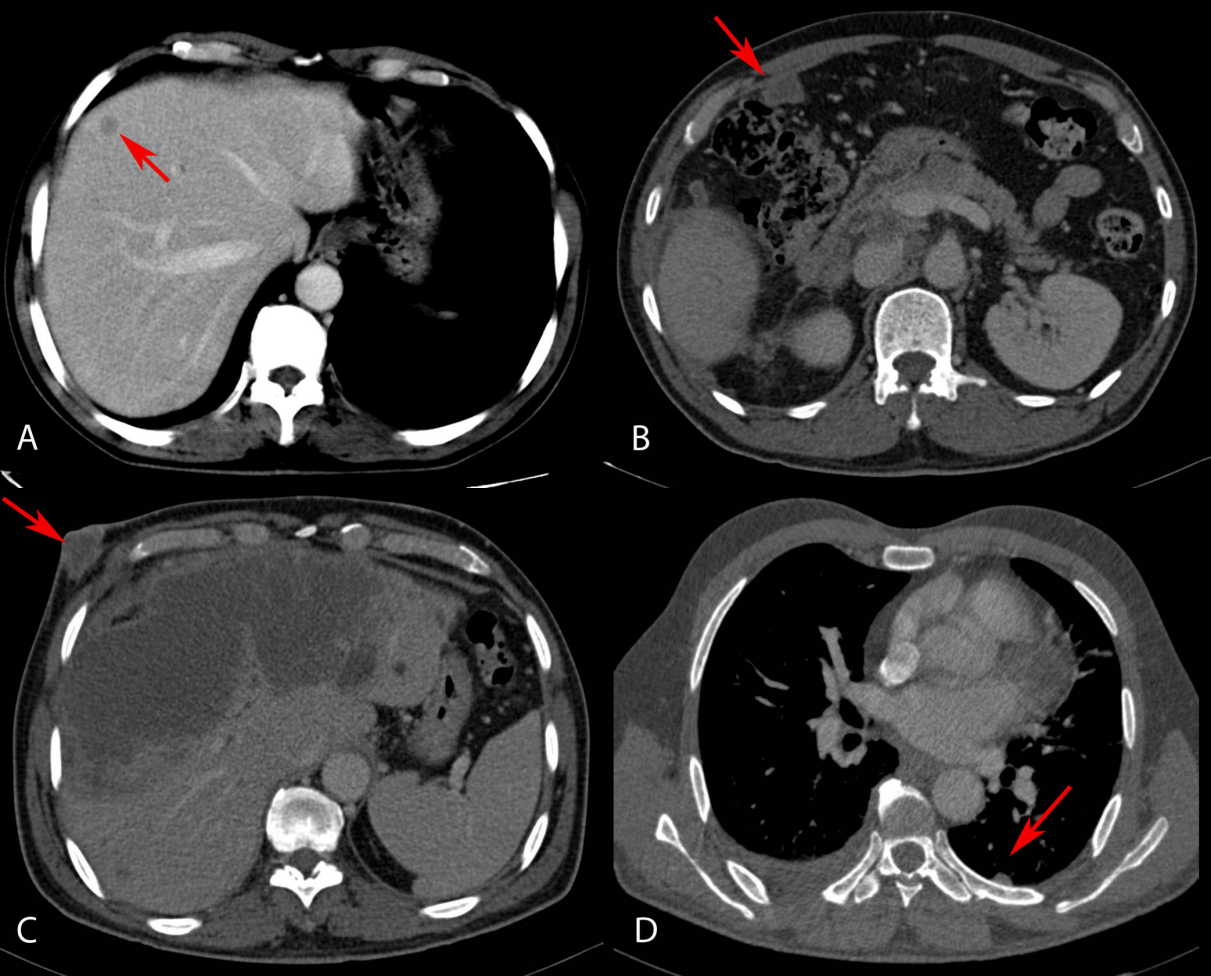
**CT images**. **A) **Hepatic metastasis (red arrow). **B) **Peritoneal metastases (red arrow). **C) **Liver infiltration, hilum adenopathies, and subcutaneous nodule at the entrance of the right laparoscopy trocar (red arrow). **D) **Nodular metastases in left pleura (red arrow) and right pleural effusion.

Nine months later, he presented with jaundice and pain in the right hypochondrium. A new CT showed right pleural effusion with nodular lesions on both pleuras, retroperitoneal and right axillary adenopathies, liver masses, peritoneal dissemination and subcutaneous nodules on the chest wall and, at the entrance of the right laparoscopy trocar, that were described as metastases (Figure 2). The patient was placed on a palliative care program which lasted for one month until his death.

## Discussion

Gallbladder carcinoma is the fifth most common malignant neoplasm of the digestive tract, adenocarcinoma being the most frequent histological type [2]. The presence of signet-ring cell proliferation accounts for a highly aggressive pathology, with only a few cases reported [8].

SRCC can arise from virtually any organ but most are from the stomach, breast, and colon [3,9]. Regardless of the tissue origin, SRCCs frequently metastasize to peritoneal surfaces, regional lymph nodes, ovaries and lungs [9].

Immunohistochemical staining is useful to determine the origin and malignant potential of the signet-ring cells. Gastric SRCC is positive for CK7, CK20 and MUC2 and negative for MUC1. Breast SRCC are mostly CK7-, MUC1-and estrogen-positive and CK20-negative. Colon SRCC are usually CK20- and MUC2-positive and CK7- and MUC1-negative [9-11]. Non-neoplastic signet-ring cells exhibit E-cadherin but no p53 mutation [5]. In our case, immunohistochemical staining of the neoplasm showed p53 mutation and CK7 positive results, with CK20 and estrogen negative results, thus confirming its malignancy and ruling out breast, colon and stomach as the SRCC origin. Therefore, the consistent histological findings (Diastase-PAS), immunohistochemical staining (eliminating the most common primary SRCC neoplasms: stomach, breast and colon) and in the absence of other primary neoplasms in the imagining studies, the patient was diagnosed with gallbladder SRCC.

Obtaining a complete medical history and performing radiological studies are the first steps in the diagnosis of possible metastatic SRCC, even before conducting immunohistochemical studies. In the case of our patient, clinical data, histology, radiology and an extensive autopsy ruled out the possibility of metastasis and non-neoplastic signet-ring cell changes.

As long as GC is known as an aggressive neoplasm, early detection and radical surgery are the best treatments. According to several series, such as those published by Kondo [12] in Japan and Dixon [13] in the US, radical surgery was proven to increase the survival rate of GC patients, becoming the most appropriate surgical option whenever possible.

Survival and prognosis of GC patients are improved by an early diagnosis; unfortunately its clinical characteristics appear at an advanced stage, so the more characteristics that are observed the poorer the prognosis. About one percent of all laparoscopic cholecystectomies present a focus of GC as an incidental finding [6].

It has been reported that the most important factor in determining the increase of survival in these patients is a negative surgical margin [13]; on the other hand, an intra-operative perforation of the gallbladder decreases survival [14]. Surgical dissemination appears to be a risk factor for peritoneal metastases. Therefore when a gallbladder carcinoma is suspected, a vinyl bag is used to wrap the specimen and prevent its spread. Dissemination by trocars used in the laparoscopy has been suspected, because they can spread cells through the abdominal wall entrance when they are removed, but this hypothesis is still questionable [15].

Although the procedure mentioned above were followed during the surgery of this patient, it is necessary to highlight the infiltrative behavior of this subtype of neoplasm (SRCC) with frequent local and distant metastases.

Tetsyri reported a case of SRCC which over-expressed c-myc oncogene and reported that a specific monoclonal antibody with reactivity against gallbladder is being studied [16]. Karabulut has also reported that signet-ring cells resemble the histology seen in the stomach SRCC and that chemotherapies such as cisplatin could be useful [8]. This is also reported by Shikata, who achieved significant positive results [17].

## Conclusion

The determination of the neoplastic or non-neoplastic origin of signet-ring cells is required to determine whether to start treatment. It is also important to recognize the origin of the primary tumor in order to optimize treatment.

The proliferation of signet-ring cells in gallbladder adenocarcinoma worsens the patient's prognosis. With only a few cases reported and an apparently ineffective classic line of treatment, we believe that more research about the biology of this cell line should be encouraged in order to modify the chemotherapy or to add biological therapy.

## Consent

Written informed consent was obtained from the patient's next-of-kin for publication of this case report and any accompanying images. A copy of the written consent is available for review by the Editor-in-Chief of this journal.

## Competing interests

The authors declare that they have no competing interests.

## Authors' contributions

FB, JS, GA, AR, MB and FZ have made substantial contributions to the conception, design, acquisition and interpretation of data; LP performed the histological examination of the gallbladder; NR has been involved in drafting the manuscript and revising it critically for important intellectual content. All authors read and approved the final manuscript.
